# Modeling of a Soft-Rigid Gripper Actuated by a Linear-Extension Soft Pneumatic Actuator

**DOI:** 10.3390/s21020493

**Published:** 2021-01-12

**Authors:** Peilin Cheng, Jiangming Jia, Yuze Ye, Chuanyu Wu

**Affiliations:** Faculty of Mechanical Engineering & Automation, Zhejiang Sci-Tech University, Hangzhou 310018, China; 202010601002@mails.zstu.edu.cn (P.C.); 201920501062@mails.zstu.edu.cn (Y.Y.); cywu@zstu.edu.cn (C.W.)

**Keywords:** static gripping system, soft-rigid gripper, soft pneumatic actuator, gripping force analysis, fingertip displacement analysis

## Abstract

Soft robot has been one significant study in recent decades and soft gripper is one of the popular research directions of soft robot. In a static gripping system, excessive gripping force and large deformation are the main reasons for damage of the object during the gripping process. For achieving low-damage gripping to the object in static gripping system, we proposed a soft-rigid gripper actuated by a linear-extension soft pneumatic actuator in this study. The characteristic of the gripper under a no loading state was measured. When the pressure was >70 kPa, there was an approximately linear relation between the pressure and extension length of the soft actuator. To achieve gripping force and fingertip displacement control of the gripper without sensors integrated on the finger, we presented a non-contact sensing method for gripping state estimation. To analyze the gripping force and fingertip displacement, the relationship between the pressure and extension length of the soft actuator in loading state was compared with the relationship under a no-loading state. The experimental results showed that the relative error between the analytical gripping force and the measured gripping force of the gripper was ≤2.1%. The relative error between analytical fingertip displacement and theoretical fingertip displacement of the gripper was ≤7.4%. Furthermore, the low damage gripping to fragile and soft objects in static and dynamic gripping tests showed good performance of the gripper. Overall, the results indicated the potential application of the gripper in pick-and-place operations.

## 1. Introduction

In a static gripping system, fragile and soft objects such as eggs, cakes, strawberries, etc., are often damaged due to excessive gripping force or large deformation. Low damage gripping to these objects is a challenge [1]. Although the traditional rigid grippers are widely used in industry or agriculture fields, some urgent issues still exist. In general, the low reliability of the precise actuation unit and the complex rigid transmission mechanism of the rigid gripper is challenging in the environment, such as a small narrow space and underwater [2,3,4]. The gripping force of the rigid grippers is precisely controlled when gripping fragile objects such as bulbs and eggs [5,6]. In contrast, when gripping soft objects such as cakes, strawberries, etc., both the gripping force and the fingertip displacement of the fingertip deep-going into the object surface must be precisely controlled. Thus, the control strategy of the rigid gripper needs to be more complicated [7].

To resolve the above issues, an increasing number of researchers have turned their attention to soft grippers, which have promising advantages with excellent flexibility [8], high environmental adaptability [9], man–machine safety [10], and low manufacturing cost as well as easy manipulation [11,12]. Compared to the soft actuators, such as those that are tendon driven using cables or shape memory alloy [13,14], electrically driven using electroactive polymers [15,16], or thermally driven using hydrogels [17,18], soft pneumatic actuators are widely used. These soft pneumatic actuators can achieve high bearing capacity and fast responses, such as a fiber-reinforced soft actuator [19,20,21] and a bellows-type soft actuator [22,23,24]. A variety of soft grippers actuated by soft pneumatic actuators have been developed, which can grip not only regular-shaped objects such as cubes and cylinders, but also irregular-shaped objects such as keys, mice, and scissors [25,26,27]. Especially, a printable soft pneumatic gripper with integrated bend sensing has been developed to grip crops such as tomato [28].

Nevertheless, the kinematics model and dynamic model of the soft gripper are difficult to establish. Furthermore, the gripping force and fingertip displacement causing damage to objects are difficult to quantitatively analyze [24] and accurately control [29,30]. Therefore, sensors are necessary in controlling a soft gripper. At present, the types of sensors used on soft grippers include resistive sensors [28,31,32,33], capacitive sensors [34,35], flexible electronics [36], strain sensitive fabrics [37], magnetic sensors [38,39], and soft pneumatic sensors [40,41]. As previous studies described, most of the above sensors are directly attached to the surface of the finger or manufactured together. Due to the poor performance of fingertip displacement measurement, it is difficult to measure the grasping force and fingertip displacement at the same time. When the soft gripper grips fragile objects such as light bulbs, eggs, etc., the broken objects will easily damage the sensor. The sensor is normally integrated on the finger and contacted with the objects directly. When the soft gripper grips soft objects such as cakes, strawberries, etc., the surface of the soft sensor is easily contaminated and is difficult to clean or replace.

The reduction of stiffness in the soft gripper, in which the fingers of gripper should behave rigidly, may decrease the overall performance of the soft grippers. It reduces the overall grasp stiffness [42] and the payload and resistive force. To address these challenges, Zhong H et al. proposed a hybrid underwater manipulator (UWM) to perform underwater operations [43]. The UWM was composed of several fiber-reinforcement soft pneumatic actuators and rigid components to increase stiffness and improve the gripping performance of the manipulator. However, the controller based on sEMG mapping could just control the posture of the manipulator, and the control of grasping force and fingertip displacement of the manipulator was not within the scope of the research. Takizawa, T. et al. developed a rigid surgical grasper actuator by a soft pneumatic actuator which had a built-in strain gauge [44]. The grasping force with acceptable error was estimated by a data-driven approach. However, a rigid cylinder was needed to limit the radial expansion of the soft actuator and ensure its axial extension. Furthermore, it was a pity that the grasping force control of the surgical grasper on human tissues was not verified, and the fingertip displacement could not be controlled, which could possible cause damage to soft and vulnerable human tissues.

Based on such studies, we proposed a soft-rigid gripper actuated by a linear-extension soft pneumatic actuator used in a static gripping system. Upon inflating air into the soft actuator, it extended along a straight line and controlled the gripper to grip the object through a slider link mechanism. We presented a non-contact sensing method for gripping state estimation to achieve simultaneous control of gripping force and fingertip displacement of the gripper without sensors integrated on the finger. An angle encoder attaches to the hinge of the finger measured its rotation angle. According to the rotation angle and the kinematic of the gripper, the extension length was derived. The relation between the pressure and extension length of the soft actuator under loading state was compared with that relationship under a no-loading state to analyze the gripping force and fingertip displacement. The linear-extension characteristic of the soft actuator makes it possible for potential applications, such as replacing the linear piston (hydraulic or pneumatic) to achieve flexible linear motion.

Specifically, this study was organized as follows: Section 2 introduced the fabrication and the mechanism of the proposed soft-rigid gripper using a linear-extension soft pneumatic actuator. Section 3 detailed the control and measurement system of the gripper. Section 4 showed the characteristic of the gripper. Section 5 presented the results of the contact state estimation, gripping force, and fingertip displacement analysis, and the gripping test of the gripper. Section 6 concluded this paper.

## 2. Soft-Rigid Gripper Using a Linear-Extension Soft Pneumatic Actuator

### 2.1. Soft-Rigid Gripper

**Figure 1:**
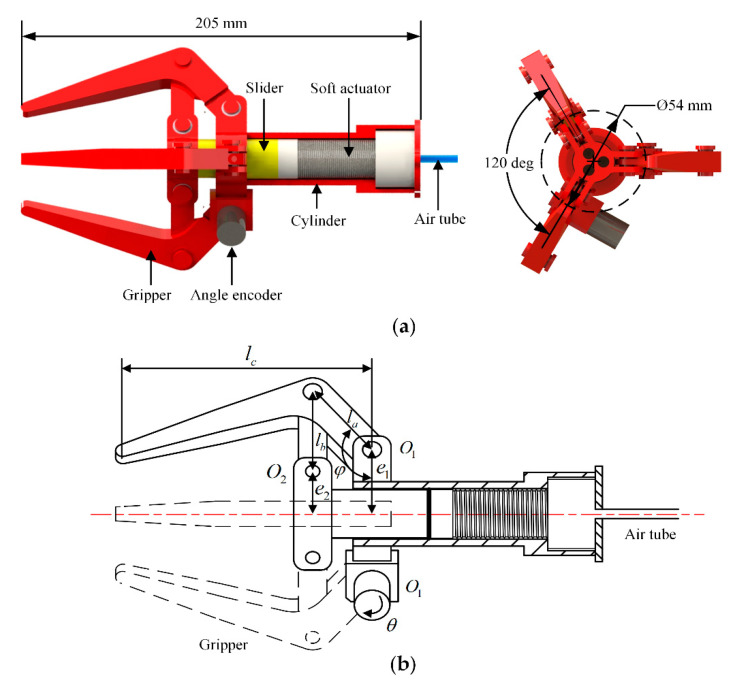
Design diagram of the soft-rigid gripper: (**a**) gripper actuated by a linear-extension soft pneumatic actuator; (**b**) schematic of the gripper.

In this study, we developed a three finger gripper with a built-in linear-extension soft pneumatic actuator and a slider link mechanism, as shown in Figure 1a. The gripper was actuated by the soft pneumatic actuator connected with a polyethylene air tube, with external and inner diameter being 4.0 and 2.0 mm, respectively. The air tube could supply compressed air from a compressor. Upon inflating air into the soft actuator, the actuator was extended to push the slider to close the fingers. After depressurization, the soft actuator pulled the slider to reset and open the gripper. Except for the soft actuator, the other components of the gripper were all rigid and were fabricated by 3D printing (PLA-F170, Stratasys). Miniature ball bearings with an outer diameter of 8 mm and inner diameter of 6 mm were installed at the hinges of the gripper to reduce the rotational friction during the opening and closing of the gripper. The angle encoder was installed at the hinge $O_{1}$ to measure its rotation angle. When the gripper was fully opened, the diameter of the inscribed circle of the three fingers was 54 mm, which determined the largest object gripped by the gripper. The height of the gripper was 205 mm. We designed the length of the connected rod *a*, *b*, and *c* shown in Figure 1b as $l_{a}=39$ mm, $l_{b}=40$ mm, and $l_{c}=105$ mm, respectively. The distances between the hinge $O_{1}$, $O_{2}$ and the centerline of the gripper were $e_{1}=27$ mm and $e_{2}=17$ mm, respectively. The angle between the connected rod *a* and the plane of $O_{1}$ was φ=140.2 deg as the fingers of the gripper are fully opened.

### 2.2. Development of the Linear-Extension Soft Pneumatic Actuator

**Figure 2:**
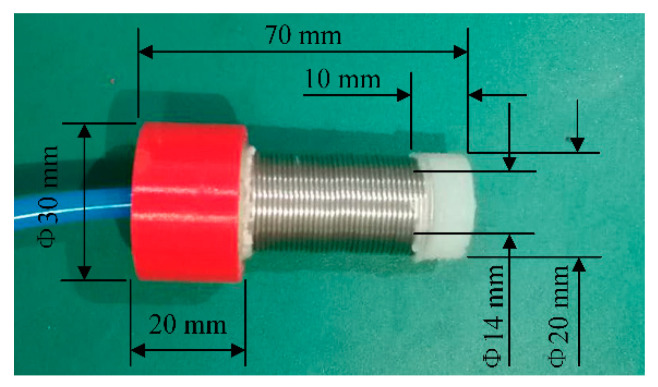
Linear-extension soft pneumatic actuator.

The developed linear-extension soft pneumatic actuator was composed of a metal spring wound on the outer wall of the cylindrical silicone cavity (Figure 2). The spring restrained the radial expansion of the soft actuator and made it extend in the axial direction during inflation. The rigid joint fabricated by 3D printing (PLA) was fixed on the air inlet end of the silicone cavity to facilitate the connection between the soft actuator and the gripper. The air tube was inserted into the silicone cavity through the rigid joint. The length of the soft actuator was 70 mm. The outer and inner diameters of the cylindrical silicone cavity were 20 and 14 mm, respectively. The specifications of the spring are shown in Table 1.

**Figure 3:**
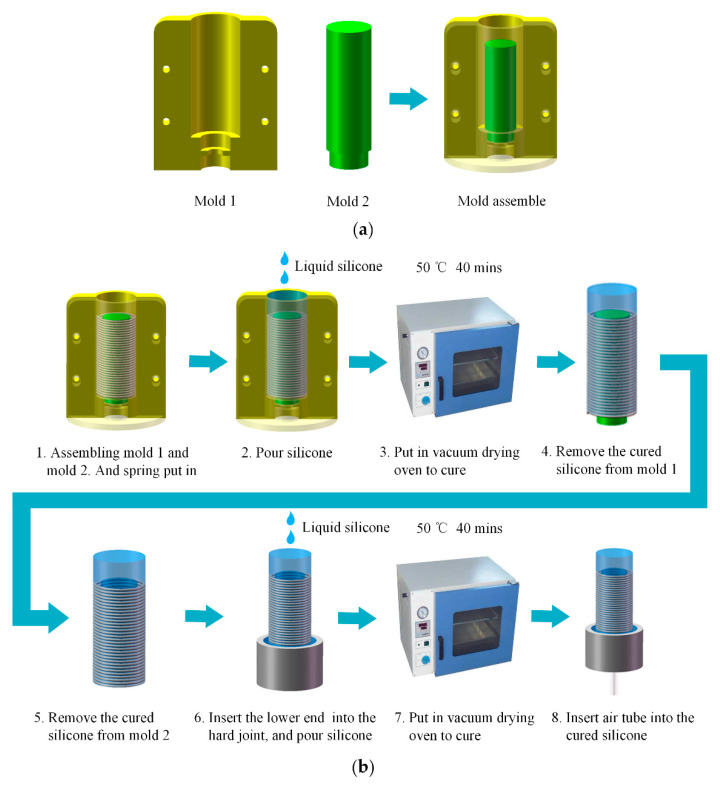
Fabrication of the soft actuator: (**a**) casting molds of the soft actuator; (**b**) fabrication procedure of the soft actuator.

In order to make the soft actuator extend along a straight-line during inflation, the fabrication process was under two conditions: (i) the outer wall of the cylindrical silicone cavity had uniform thickness; (ii) the centerline of the spring coincided with the centerline of the cylindrical silicone cavity. To satisfy the above two conditions, the casting molds of the soft actuator were designed (Figure 3a). A couple of molds (mold 1) were assembled into an outer mold with a cylindrical cavity whose diameter was equal to the outer diameter of the spring. The inner mold 2 was inserted into the positioning hole at the bottom of the cavity to complete the mold assembly. Both mold 1 and mold 2 were fabricated by 3D printing (PLA).

**Figure 4:**
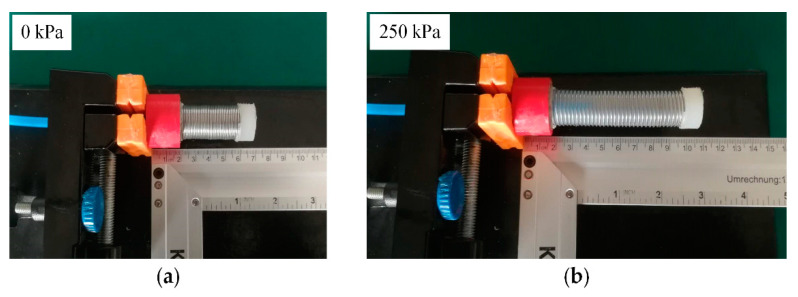
Deformation of the soft actuator: (**a**) initial state at 0 kPa; (**b**) pressurized state with 250 kPa.

The fabrication process of the soft pneumatic actuator is shown in Figure 3b. Firstly, the spring was inserted into the assembled mold. Furthermore, the degassed liquid silicone (Dragon Skin 20, Smooth-on Inc., Easton, PA, USA) was poured. Then, we put the mold into the vacuum drying oven to cure the silicone for 2 h at 60 °C. After curing, the silicone cavity was released from the molds, the lower end of the cavity was inserted into the rigid joint, and the degassed liquid silicone was poured into the gap between rigid joint and the silicone cavity. Then, we put them into the vacuum drying oven to cure the silicone for 2 h at 60 °C. Further, the sealing of the bottom of the silicone cavity was completed after curing. At last, an air tube was inserted into the silicone cavity through the rigid joint to complete the fabrication of the soft actuator. Figure 4 shows the deformation of the soft actuator. Specifically, Figure 4a shows the actuator in initial condition, and Figure 4b shows the actuator extended in pressurized condition at 250 kPa. Furthermore, the soft actuator can be extended in a straight-line during pressurization.

## 3. Control and Measurement System

### 3.1. Experiment Apparatus

**Figure 5:**
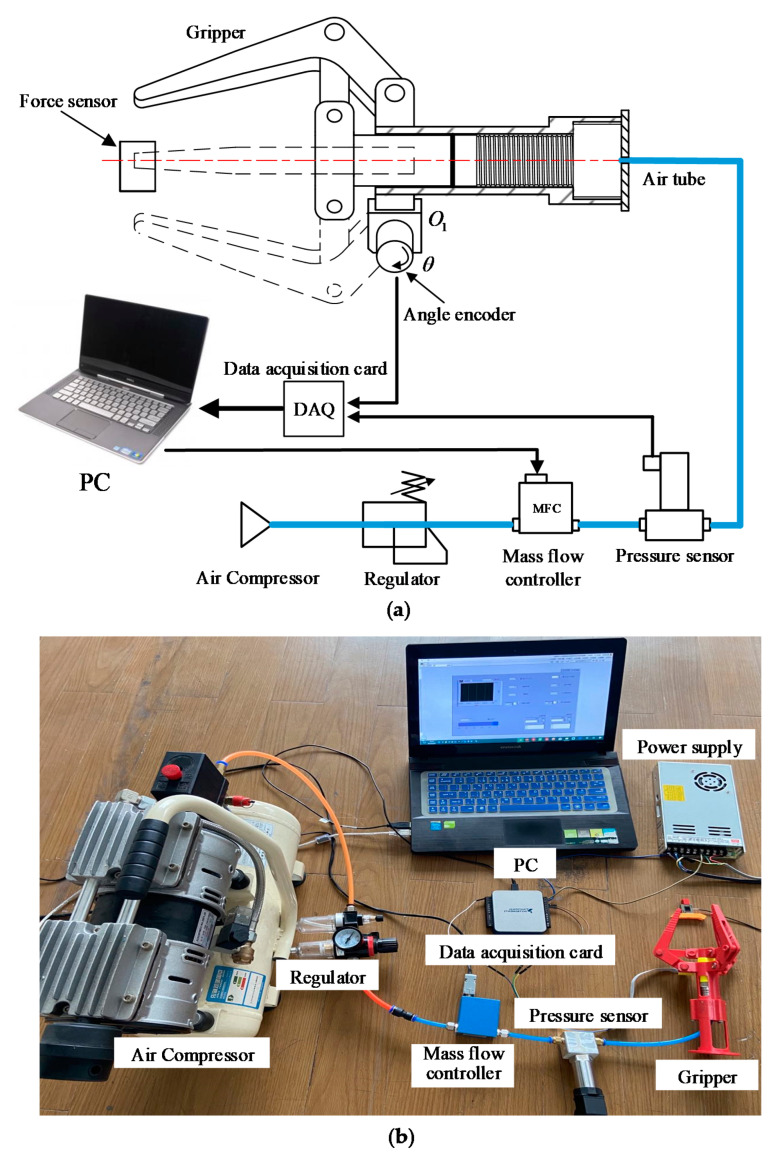
Experimental apparatus for control and measurement system of the gripper: (**a**) schematic of control and measurement system; (**b**) control and measurement system used in the validation test.

Figure 5 shows the experiment apparatus for characteristics evaluations. NI LabView software was applied on the PC to read the pressure and rotation angle sampled by the data acquisition card (NI USB-6001) from the pressure sensor and angle encoder. Then, the control signal was sent to the Mass Flow Controller (MFC300, Aitoly Electronic Technology Co., Ltd., Suzhou, China) to control the air flow. The pressure sensor (MKI-P300, Meacon Automation Technology Co., Ltd., Hangzhou, China) measured the pressure of the soft actuator. The angle encoder (QY1503-CDZ5E, Accnt Electronics Co., Ltd., Shanghai, China) with a resolution of 4096 measured the rotation angle of the hinge $O_{1}$. A force sensor (DYLY-108-10, DaYang Sensing System Engineering Co., Ltd., Bengbu, China) with the range (0∼10 N) measured the gripping force of the gripper. This sensor was used for the gripping force estimation experiment.

### 3.2. Control Method

**Figure 6:**
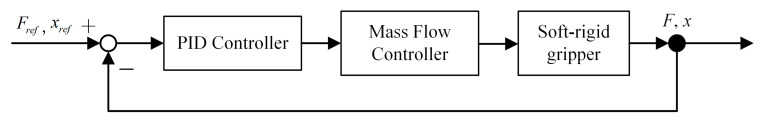
Block diagram of the control method in control and measurement system of the gripper.

Figure 6 shows the block diagram of the control method in control and measurement system. A cascade controller consisting of a loop was adopted to control the gripping force *F* and fingertip displacement *x* of the gripper. Based on the error between the input and output of the system, PID was performed to control the flow of the Mass Flow Controller. Then, the increase speed of the pressure and closing speed of the fingers were adjusted. The gripping force *F* and fingertip displacement *x* of the gripper could be calculated from the pressure of the soft actuator in Section 5.

## 4. Characteristics of the Gripper

Generally, the gripper only damages the targeted object during the gripping process; therefore, we focused on measuring and analyzing the characteristic of the gripper during pressurization.

In detail, we established the relationship between the pressure *P* and the extended length *s* of the soft actuator in no loading state of the gripper. The soft actuator was inflated until the gripper was fully closed. The maximum flow of the mass flow controller was 100 mL/min (ANR). The sampling rate of the data acquisition card was 1000 Hz. It synchronously sampled the pressure *P* of the soft actuator and the rotation angle θ of the hinge $O_{1}$. This trial was carried out ten times under the same conditions to confirm the repeatability. During the closing of the gripper, the kinematic analysis of the gripper showed the relation between the rotation angle θ of the hinge $O_{1}$ and the extended length *s* of the soft actuator as follows:

(1) $$s=l_{a}[\sin(\varphi -\theta )-\sin\varphi ]+\sqrt{l_{b}^{2}-{\left[(e_{1}-e_{2})-l_{a}\cos(\varphi -\theta )\right]}^{2}}$$

where $l_{a}$, $l_{b}$ and $l_{c}$ are the length of the connected rod *a*, *b*, and *c* of the gripper, $e_{1}$ and $e_{2}$ are the distance between the hinge $O_{1}$, $O_{2}$ and the centerline of the gripper, and φ is the angle between the connected rod *a* and the plane of $O_{1}$, as shown in Figure 1b.

**Figure 7:**
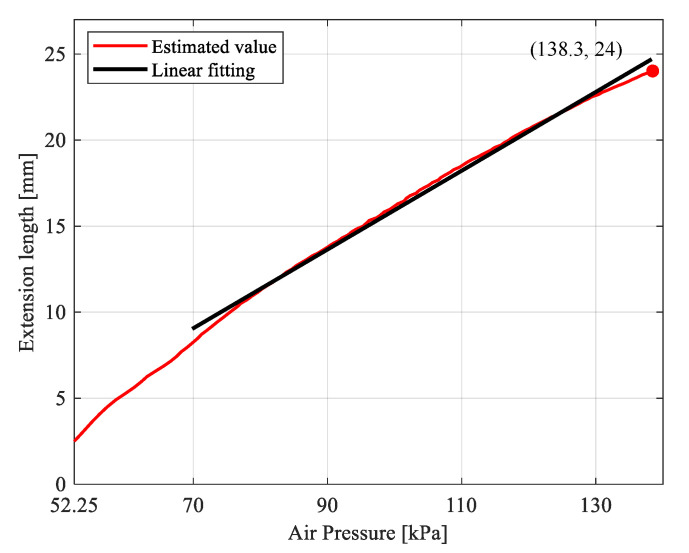
Relationship between the pressure and the extension length of the soft actuator.

The rotation angle θ of the hinge $O_{1}$ sampled by the data acquisition card was converted into the extension length of the soft actuator through Equation (1). The relationship between the extended length *s* and the pressure *P* of the soft actuator was obtained, as shown by the red line in Figure 7. The gripper was fully closed with the pressure of 138.3 kPa and the extension length of 24 mm. In the process of inflating the soft actuator, when the pressure was <52.25 kPa, the soft actuator did not extend due to the inability to overcome the sliding friction between the slider and the cylinder and the rotational friction of the hinges of the gripper. In the process of increasing the pressure from 52.25 to 70 kPa, the extension process of the soft actuator was unstable due to the overcoming of friction.

Therefore, when the pressure was >70 kPa, the relationship between the pressure and the extension length of the soft actuator was approximately linear as the high elasticity of the spring. Furthermore, the nonlinear extension characteristic of the silicone cavity was not obvious. However, when the pressure was >130 kPa, the nonlinear extension characteristic of the silicone cavity became extremely significant, resulting in a nonlinear extension trend of the soft actuator. Linear fitted the relation between the pressure and the extension length of the soft actuator when the pressure was >70 kPa, as shown by the black line in Figure 7. The maximum absolute value of the absolute error between the linear fitting extension length of the soft actuator and the measured extension length under the corresponding pressure was 0.82 mm. The equation of the linear fitting curve was as follows:

(2) s=0.229P−6.954

## 5. Validation Experiments

### 5.1. Contact State Estimation

**Figure 8:**
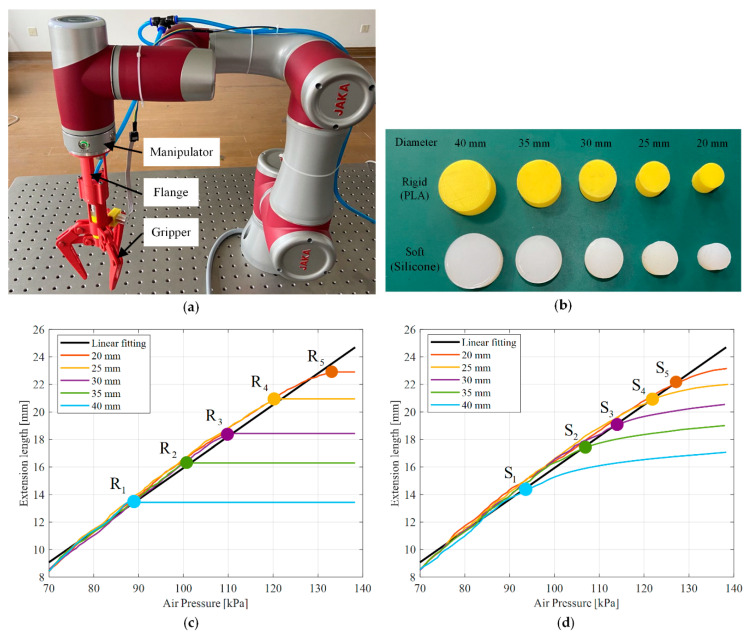
Contact state estimation of the gripper during validation test: (**a**) fixed gripper on the end of manipulator; (**b**) rigid and soft cylindrical objects; (**c**) relationship between pressure and extension length of the gripper gripping rigid cylinders; (**d**) relationship between pressure and extension length of the gripper gripping soft cylinders.

In the case of no sensor integrated, the estimation of the contact state between the fingers and the object is the premise of the grasping force and fingertip displacement analysis. Contact extension length and contact pressure are the extension length and pressure of the soft actuator when the fingers are in contact with the object. They were estimated from the linear fitting relationship between the pressure and extension length shown by the black line in Figure 7. In this experiment, the gripper was fixed on the manipulator with a flange as shown in Figure 8a. The gripper gripped the rigid and soft cylindrical objects with diameters of 40, 35, 30, 25, and 20 mm in Figure 8b, respectively. The rigid cylindrical objects were fabricated by 3D printing (PLA). The soft cylindrical objects with a cavity inside were fabricated by degassed liquid silicone (Dragon Skin 20) by mold casting.

Figure 8c,d show the relationship between the pressure and the extension length of the soft actuator when the gripper gripped the rigid and soft cylindrical objects, respectively. The linear fitting relation between the pressure and the extension length under no loading state of the gripper (the black line in Figure 7) was also plotted for comparison. As shown in Figure 8c, compared with the black line, the relationship between the pressure and extension length with the gripper gripped the rigid cylindrical objects with diameters of 40, 35, 30, 25, and 20 mm displaced at points $R_{1}$, $R_{2}$, $R_{3}$, $R_{4}$, and $R_{5}$, respectively. These points ($R_{1}$, $R_{2}$, $R_{3}$, $R_{4}$, and $R_{5}$) were the contact points between the fingers and the rigid cylindrical objects. As shown in Figure 8d, compared with the black line, the relationship between the pressure and extension length when the gripper gripped the soft cylindrical objects with diameters of 40, 35, 30, 25, and 20 mm displaced at points $S_{1}$, $S_{2}$, $S_{3}$, $S_{4}$, and $S_{5}$, respectively. These points ($S_{1}$, $S_{2}$, $S_{3}$, $S_{4}$, and $S_{5}$) were the contact points between the fingers and the soft cylindrical objects.

However, the relationship between the pressure and extension length of the soft actuator (Figure 8d) was different. When the gripper gripped the soft cylindrical object, the soft actuator slowly extended and gradually became nearly unchanged with the increase of the pressure after the fingers were in contact with the object. The reason was that with increase of the pressure, the soft actuator slowly actuated the gripper to be closed gradually to deform the soft cylindrical object after the fingers contacted with the object. As the deformation of soft cylindrical object increased, its ability to resist deformation was gradually improved. Therefore, the extension of the soft actuator gradually became nearly unchanged with the increase of the pressure.

When the gripper gripped the rigid or soft cylindrical objects, we proposed the non-contact sensing method to estimate the contact pressure and contact extension length of the soft actuator with respect to the corresponding contact point. The pressure and extension length of the soft actuator sampled by the data acquisition card were continuously detected. Further, the absolute values of the absolute errors between several extension length values and corresponding extension lengths under a no-loading state (the black line in Figure 8c,d were greater than 0.82 mm. The 0.82 mm was the maximum absolute value of the absolute error between the linear fitting extension length and measured extension length under the corresponding pressure in Figure 7. It might be caused by the fingers had been contact with the object. Then, we considered the contact pressure and contact extension length of the soft actuator were just the pressure and extension length sampled by the data acquisition card ahead of the compared pressure and extension length. In this case, the number of compared pressure and extension length was five.

**Figure 9:**
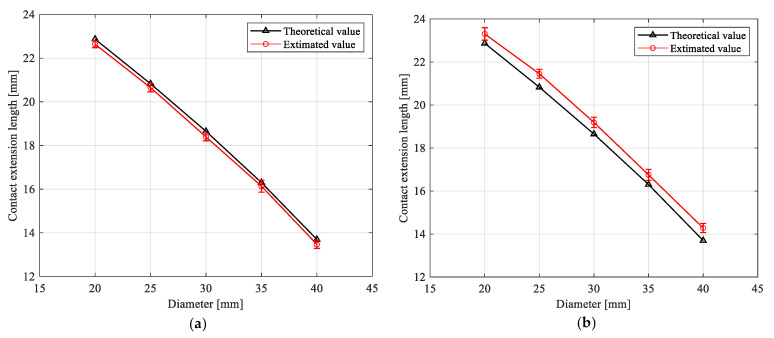
Estimation of contact extension length of the gripper. (**a**) Estimating contact extension length of gripper gripping rigid cylindrical objects, and (**b**) estimating contact extension length of gripper gripping soft cylindrical objects.

The gripper gripped the rigid and soft cylindrical objects with five diameters and estimated the contact pressure and extension length by the non-contact sensing method. The gripper gripped each sized cylindrical object 10 times to confirm the repeatability of the trial. Figure 9a,b show the experimental results of contact extension length estimation of the gripper, with gripping the rigid and soft cylindrical objects, respectively. The black triangles were the theoretical contact extension length of the soft actuator, which were calculated by Equation (1). The red circles represented the average value of the estimated contact extension length, while the error bar showed the standard deviations of the estimation.

The maximum relative errors of the estimated contact extension length and theoretical contact extension length for the rigid and soft cylinder objects were 1.7% and 4.3% (Figure 9a,b), respectively. Such results demonstrated the effectiveness of non-contact sensing method. The corresponding maximum standard deviations of the estimated contact extension length were 0.27 and 0.29 mm, respectively. The small standard deviation values indicated that the contact state estimation achieved good repeatability.

When the gripper gripped the soft cylindrical objects, the estimated contact extension length was always greater than the theoretical contact extension length of the corresponding diameter cylindrical object (Figure 9b). The main reason for this phenomenon is that the contact state estimation method introduces the contact extension length estimation error, which causes the estimated contact point to lag the theoretical contact point. Therefore, we revised the analytical fingertip displacement to eliminate the estimation error of contact extension length when analyzing the fingertip displacement of the gripper gripping the soft object in Section 5.3.

### 5.2. Gripping Force Analysis

**Figure 10:**
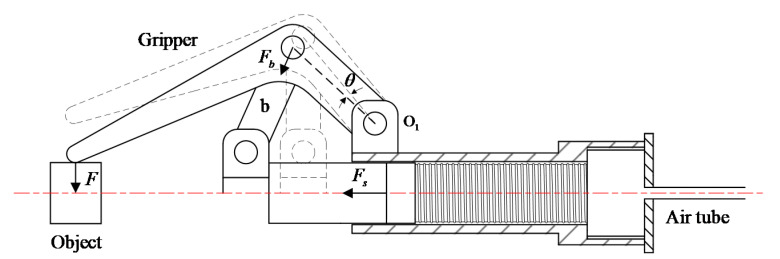
Gripping force analysis of the gripper.

The gripping force was calculated from the contact pressure of soft actuator which can be estimated from Section 5.1. In the process of the gripper gripping rigid or soft objects, all three fingers were loaded the same force. Among them, one finger was analyzed in Figure 10 (the sliding friction between the slider and the cylinder and the rotational friction of the hinges of the gripper were ignored). The plane moment balance equation of hinge $O_{1}$ is expressed as follows:

(3) $$Fl_{c}-F_{b}l_{a}\cos(\arccos(\frac{l_{a}\sin(\varphi -\theta )+s}{l_{b}})-(\varphi -\theta ))\cos\theta =0$$

where *F*, $F_{b}$, and θ denote the analytical gripping force of the gripper, the pulling force of the connected rod b to the finger, and the rotation angle of the hinge $O_{1}$. The relationship between the pulling force $F_{b}$ and the axial thrust force $F_{s}$ of the soft actuator is:

(4) $$F_{s}=3F_{b}\cos(s+l_{a}\sin\varphi -l_{a}\sin(\varphi -\theta ))$$

where *s* is the extension length of the soft actuator. The relation between the pressure *P* and the axial thrust force $F_{s}$ of the soft actuator is expressed by Equation (5).

(5) $$F_{s}=(P-P_{c})A$$

where $P_{c}$ is the contact pressure of the soft actuator and *A* is the bottom area of the cylindrical cavity of the soft actuator, which is consistent during processing. By substituting Equations (4) and (5) into Equation (3), the relationship between the analytical gripping force *F* and the pressure *P*, the contact pressure $P_{c}$ is expressed as Equation (6):

(6) $$F=\frac{(P-P_{c})Al_{a}\cos(\arccos(\frac{l_{a}\sin\varphi -l_{a}\sin(\varphi -\theta )+s}{l_{b}})-(\varphi -\theta ))\cos\theta }{3l_{c}\cos(s+l_{a}\sin\varphi -l_{a}\sin(\varphi -\theta ))}$$

**Figure 11:**
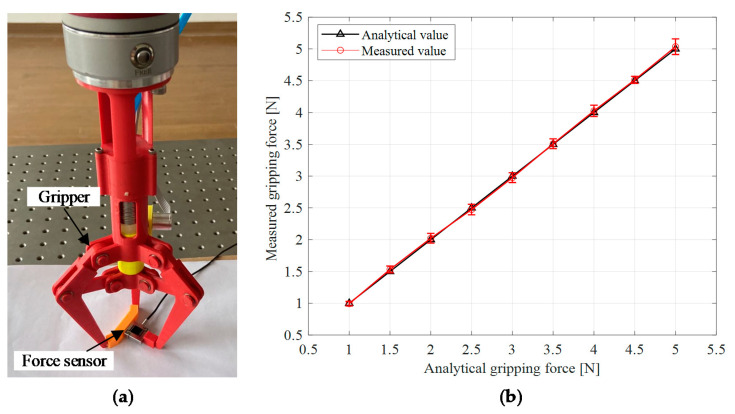
Gripping force measurement for the designed gripper: (**a**) gripping force measurement method illustration; (**b**) gripping force measurement result.

The gripper gripped the force sensor to measure the gripping force as shown in Figure 11a. In this experiment, the gripping force thresholds were from 1 to 5 N, with a step size of 0.5 N. The pressure of the soft actuator increased, until the analytical gripping force reached the gripping force threshold. Then, the gripping force measured by the force sensor was recorded. This experiment was carried out 10 times under the same conditions for each gripping force threshold to make sure the repeatability.

The relationship between the analytical grasping forces and the measured values was shown in Figure 11b. The black triangles were the analytical gripping force, while the red circles represented the average value of the measured gripping force, and the error bars showed the standard deviations of the measuring. The maximum relative error between the analytical gripping force and the average value of the measured gripping force was 2.1%, indicating that the analytical grasping force was valid. The maximum standard deviation of the measured gripping force was 0.12 N, which was fairly small and indicated that this experiment achieved good repeatability.

### 5.3. Fingertip Displacement Analysis

The fingertip displacement was calculated from the contact extension length of the soft actuator which can be estimated from Section 5.1. In the process of gripping the soft object, the kinematic analysis of the gripper shows that the relation between the analytical fingertip displacement $x_{a}$ and the rotation angle θ of the hinge $O_{1}$, as follows in Equation (7):

(7) $$x_{a}=2l_{c}\sin\frac{\theta -\theta_{ec}}{2}$$

where, $\theta_{ec}$ is the estimated rotation angle of the hinge $O_{1}$ when the fingers are in contact with the objects. It can be calculated from the estimated contact extension length of the soft actuator by Equation (1). To eliminate the estimation error of the contact extension length introduced by the non-contact sensing method in Section 5.1, the analytical fingertip displacement $x_{a}$ calculated by Equation (7) was revised. The relation between the revised analytical fingertip displacement $x_{a}$ and the rotation angle θ is as follows:

(8) $$x_{a}=2l_{c}\sin\frac{\theta -(\theta_{ec}-\theta_{r})}{2}$$

where, $\theta_{r}$ is the revised deviation of the $\theta_{ec}$, which can be calculated from the estimation error of the contact extension length of the soft actuator by Equation (1).

The gripper gripped the soft cylindrical object with the diameter of 30 mm shown as Figure 8d to estimate the fingertip displacement of the gripper by Equation (8), which was compared with the theoretical fingertip displacement $x_{t}$ calculated by the following equation:

(9) $$x_{t}=2l_{c}\sin\frac{\theta -\theta_{tc}}{2}$$

where $\theta_{tc}$ is the theoretical rotation angle of the hinge $O_{1}$ when the fingers are in contact with the soft cylindrical object with the diameter of 30 mm, which is an theoretical value.

In this experiment, the theoretical fingertip displacements were set from 1 mm to 5 mm with a step size of 0.5 mm. At each theoretical fingertip displacement, there was a corresponding rotation angle θ could be calculated by Equation (9), which was a preset angle. The pressure of the soft actuator increased gradually until the rotation angle θ reached the preset angle, that is, the theoretical fingertip displacement reached the set value. Then, the analytical fingertip displacement calculated by Equation (8) was recorded. At each theoretical fingertip displacement, this experiment was carried out 10 times under the same conditions to confirm the repeatability.

**Figure 12:**
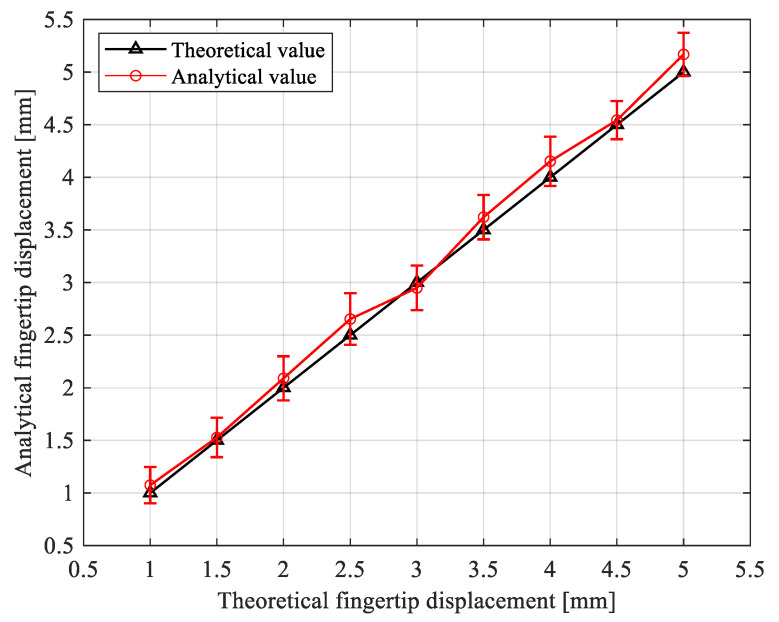
Analysis result for the fingertip displacements.

The relationship between the analytical fingertip displacements and theoretical values was shown in Figure 12. The black triangles were the theoretical fingertip displacements. The red circles represented the average value of the analytical fingertip displacements, while the error bar showed the standard deviations of the analysis. The maximum relative error between the theoretical fingertip displacement and the average value of the analytical fingertip displacement was 7.4%, which indicated that the fingertip displacement analysis based on the contact state estimation was effective. The maximum standard deviation of the analytical fingertip displacement was 0.23 mm, which indicated that this experiment obtained good repeatability.

### 5.4. Static Gripping Test

**Figure 13:**
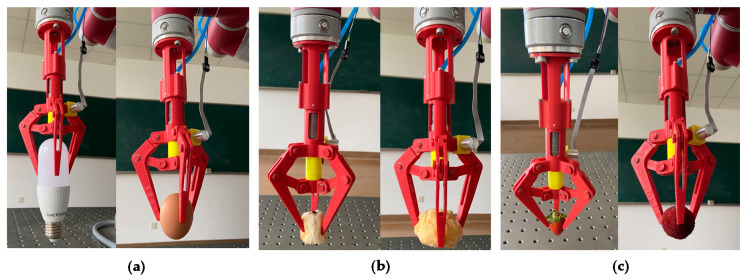
Static gripping test of the gripper for controlling gripping force and fingertip displacement. (**a**) Control the gripping force to 2 N to grip light bulb and raw egg, (**b**) control the fingertip displacement to 3 mm to grip bread and cake, and (**c**) control the gripping force to 1 N or the fingertip displacement to 1 mm to grip strawberry and bayberry.

Based on the approved analysis of the grasping force and fingertip displacement, they were controlled during the process of the gripper gripping fragile or soft objects. In this experiment, the gripper griped light bulb, raw egg, bread, cake, strawberry, and bayberry, respectively, as shown in respective Figure 13a–c. Before the gripping test, a pre-experiment was conducted to test the minimum gripping force and fingertip displacement required for the gripper to stably lift the above objects. The resulted values would be as the safe thresholds of the gripping force and fingertip displacement.

The damage form of light bulb and raw egg (fragile objects) can be defined as breaking by excessive gripping force. Thus, the gripping force was controlled during the gripping process in Figure 13a. A safe gripping force threshold (2 N) was set in the control and measurement system of the gripper. Inflated the soft actuator until the analytical gripping force reached the gripping force threshold. Then, the object was moved to a specified position by the manipulator.

The damage form of bread and cake (soft foods) is the destructive deformation caused by excessive fingertip displacement. So, the fingertip displacement was controlled during the gripping process (Figure 13b). A safe fingertip displacement threshold (3 mm) was set in the control and measurement system of the gripper. We inflated the soft actuator until the analytical fingertip displacement reached the fingertip displacement threshold. Then, the object was moved to a specified position by the manipulator.

The damage form of strawberry and bayberry is the tissue injury caused by excessive gripping force or excessive fingertip displacement. Thus, the gripping force and fingertip displacement were controlled during the gripping process (Figure 13c). Compared with light bulb and raw egg, strawberry and bayberry can bear less gripping force. Compared with bread and cake, strawberry and bayberry can bear less fingertip displacement. A safe gripping force threshold (1 N) and a safe fingertip displacement threshold (1 mm) were set in the control and measurement system of the gripper. We inflated the soft actuator until the analytical grasping force reached the gripping force threshold or the analytical fingertip displacement reached the fingertip displacement threshold. Then, the object was moved to a specified position by the manipulator.

As shown in Figure 13a–c, during the process of gripping the objects, the gripping force and fingertip displacement were respectively controlled according to the damage forms of fragile objects. The fragile objects included light bulb and raw egg, soft food (bread and cake), soft fruits (strawberry and bayberry), to achieve the low or without gripping damage. In the gripping test, neither the light bulb nor raw egg was broken with excessive grasping force. The bread and cake maintained their initial shape under a safe gripping deformation threshold. Furthermore, there was no epidermal tissue injury for both strawberry and bayberry with excessive gripping force or excessive fingertip displacement. Such results further demonstrated the effectiveness of the contact state estimation, gripping force analysis, and fingertip displacement analysis of the gripper.

### 5.5. Dynamic Gripping Test

**Figure 14:**
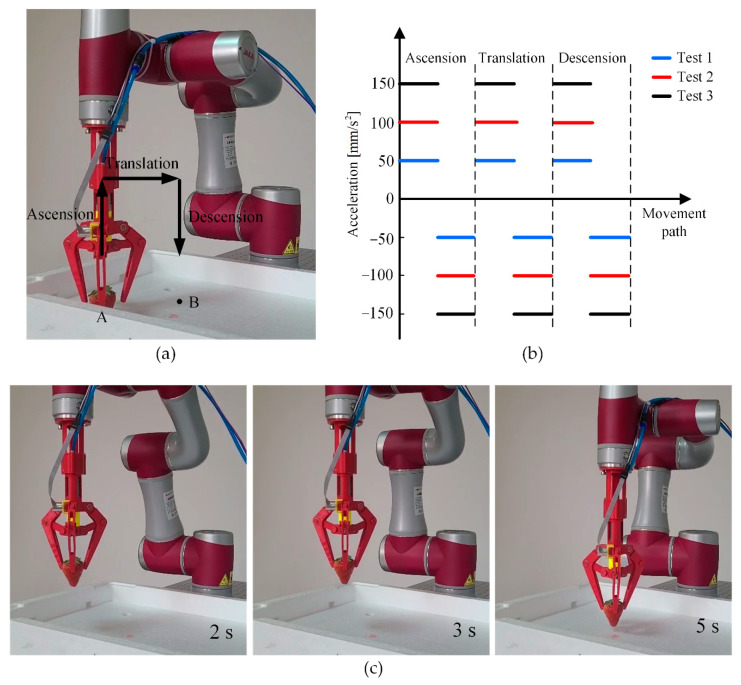
Dynamic gripping test of the gripper. (**a**) The movement path of the gripper in the dynamic gripping test was divided into ascension, translation, and descension, (**b**) the gripper had two movement states at each movement path, (**c**) The image snapshots of the dynamic gripping of the gripper under different times at the acceleration of 150 mm/s^2^.

**Figure 15:**
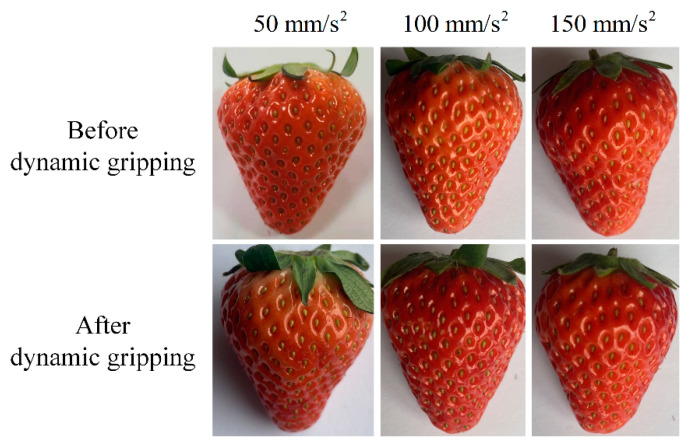
The comparison of the damage of the strawberry before and after the dynamic gripping.

In addition to proving the static gripping performance of the gripper in Section 5.4, we conducted an experiment to further demonstrate that the gripper has certain dynamic gripping ability for vulnerable objects in Figure 14. As shown in Figure 14a, the gripper fixed at the end of the manipulator gripped strawberries with a safe gripping force (1 N), then, the manipulator moved the strawberry from point A to point B. The movement path of the manipulator was divided into ascension, translation, and descension. Limited by the movement space of the manipulator, the movement distance of the manipulator at each movement path was 120 mm. As shown in Figure 14b, at each movement path, the manipulator was set to have two movement states: uniform acceleration and uniform deceleration, and the acceleration values of the two movement states were equal. In addition, the acceleration values of each movement path were set to be same. Moreover, in order to observe the damage of the gripper to the strawberry under different accelerations, the accelerations were set from 50 to 150 mm/s^2^, with a step of 50 mm/s^2^. At each acceleration, the motion of the manipulator was record by a camera (Movie S1 in the Supplementary Material). The image snapshots of the manipulator taken at 2, 3, and 5s at the acceleration of 150 mm/s^2^ were shown in Figure 14c. Then, under different accelerations, the damage of the strawberry before and after the movement of the manipulator were compared in Figure 15. As shown in Figure 15, at each acceleration, the strawberry had almost no damage before and after the movement of the manipulator, which also demonstrated the dynamic gripping performance of the gripper for vulnerable objects.

## 6. Conclusions and Future Work

We proposed a soft-rigid gripper, which was actuated by a linear-extension soft pneumatic actuator composed of a metal spring wound on the outer wall of a cylindrical silicone cavity. For fingers without an integrated sensor, a non-contact sensing method for gripping state estimation was used to estimate the contact pressure and contact extension length of the soft actuator and analyze the gripping force and fingertip displacement of the gripper. The experiment results showed that relative error between the estimated contact extension length and theoretical contact extension length of the soft actuator was ≤4.3%. The relative error between the analytical gripping force and the measured gripping force of the gripper was ≤2.1%. Furthermore, the relative error between the analytical fingertip displacement and theoretical fingertip displacement of the gripper was ≤7.4%. Further, by controlling the gripping force and fingertip displacement, the gripper achieved low or without gripping damage to various fragile objects, soft food, and berry fruits in a static gripping test. Furthermore, the gripper achieved low gripping damage to the strawberry in a dynamic gripping test. These results demonstrated the effectiveness of the approved estimation method.

In general, this work made contributions, providing a useful tool for researchers working with a soft-rigid gripper, especially for those interested in using a non-contact sensing method to estimate the gripping state of the gripper. In the future, we will apply the gripper for fruit picking in fields and food processing in production. Furthermore, we plan to perform finite element modeling (FEM)-based simulations to analyze the properties of the linear-extension soft pneumatic actuator. Moreover, we will design soft-rigid embedded grippers with multiple variable stiffness fingers to target complex manipulation issues.

## Figures and Tables

**Table 1:** Specification of the spring.

Spring Parameter	Value
Material	SUS304WPB
Wire diameter	1.0 mm
Outside diameter	20.0 mm
Pitch	1.0 mm

## Data Availability

The data presented in this study are available on request from the corresponding author.

## Supplementary Materials

The following are available online at https://www.mdpi.com/1424-8220/21/2/493/s1, Movie S1: Dynamic gripping test processes under different accelerations.

## Abbreviations

Contact pressure	Pressure of the soft actuator when the fingers are in contact with the object
Contact extension length	Extension length of the soft actuator when the fingers are in contact with the object
Theoretical contact extension length	Theoretical value of the contact extension length
Estimated contact extension length	Estimated value of the contact extension length
Contact angle	Rotation angle of the hinge $O_{1}$ when the fingers are in contact with the object
Theoretical contact angle	Theoretical value of the contact angle
Estimated contact angle	Estimated value of the contact angle
Analytical gripping force	Analytical value of the gripping force calculated by Equation (6)
Fingertip displacement	Displacement of the fingertip deep-going into the object surface
Analytical fingertip displacement	Analytical value of the fingertip displacement calculated by Equation (8)
Theoretical fingertip displacement	Accurate value of the fingertip displacement
